# *N*-(2-Amino­pyridin-3-yl)-4-methyl-*N*-(4-methyl­phenyl­sulfon­yl)benzene­sulfonamide

**DOI:** 10.1107/S1600536812010872

**Published:** 2012-03-21

**Authors:** Abu Taher, Vincent J. Smith

**Affiliations:** a Department of Chemistry and Polymer Science, University of Stellenbosch, Private Bag X1, Matieland 7602, South Africa

## Abstract

The title compound, C_19_H_19_N_3_O_4_S_2_, was prepared by the reaction of 2,3-diamino­pyridine with tosyl chloride in a mixture of dichloro­methane–pyridine as solvent. In the crystal, mol­ecules associate *via* pairs of N—H⋯N hydrogen bonds, forming a centrosymmetric eight-membered {⋯HNCN}_2_ synthon. The dihedral angles between the amino­pyridine ring and the tosyl benzene rings are 50.01 (6) and 32.01 (4)°.

## Related literature

For the synthesis of related compounds, see: Schetty (1969 ▶); Dubey & Kumar (2000 ▶). For background to the application of ring-closing metathesis (RCM) on substrates protected with sulfonamide groups, see: Yadav *et al.* (2011 ▶); Morgans *et al.* (2009 ▶); van Otterlo *et al.* (2004 ▶). For graph-set notation, see: Bernstein *et al.* (1995 ▶).
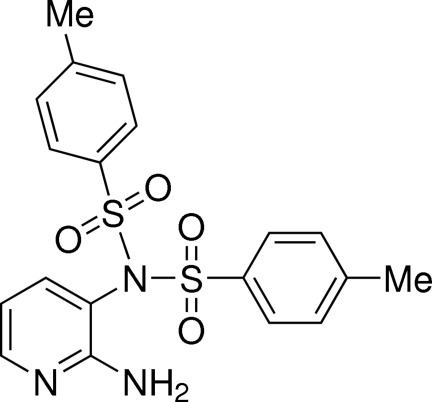


## Experimental

### 

#### Crystal data

C_19_H_19_N_3_O_4_S_2_
*M* *_r_* = 417.49
Triclinic, 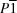

*a* = 8.6343 (15) Å
*b* = 9.6486 (17) Å
*c* = 12.701 (2) Å
α = 111.324 (2)°
β = 90.109 (2)°
γ = 98.097 (2)°
*V* = 974.2 (3) Å^3^
*Z* = 2
Mo *K*α radiation
μ = 0.30 mm^−1^
*T* = 102 K
0.25 × 0.25 × 0.25 mm

#### Data collection

Bruker APEXII CCD diffractometer
Absorption correction: multi-scan (*SADABS*; Bruker, 2009 ▶) *T*_min_ = 0.928, *T*_max_ = 0.928
11658 measured reflections
4644 independent reflections
4381 reflections with *I* > 2σ(*I*)
*R*_int_ = 0.015

#### Refinement

*R*[*F* ^2^ > 2σ(*F* ^2^)] = 0.031
*wR*(*F* ^2^) = 0.086
*S* = 1.03
4644 reflections
256 parameters
H-atom parameters constrained
Δρ_max_ = 0.47 e Å^−3^
Δρ_min_ = −0.35 e Å^−3^

### 

Data collection: *APEX2* (Bruker, 2009 ▶); cell refinement: *SAINT* (Bruker, 2009 ▶); data reduction: *SAINT*; program(s) used to solve structure: *SHELXS97* (Sheldrick, 2008 ▶); program(s) used to refine structure: *SHELXL97* (Sheldrick, 2008 ▶); molecular graphics: *X-SEED* (Barbour, 2001 ▶); software used to prepare material for publication: *X-SEED*.

## Supplementary Material

Crystal structure: contains datablock(s) I, global. DOI: 10.1107/S1600536812010872/tk5064sup1.cif

Structure factors: contains datablock(s) I. DOI: 10.1107/S1600536812010872/tk5064Isup2.hkl

Supplementary material file. DOI: 10.1107/S1600536812010872/tk5064Isup3.cml

Additional supplementary materials:  crystallographic information; 3D view; checkCIF report

## Figures and Tables

**Table 1:** Hydrogen-bond geometry (Å, °)

*D*—H⋯*A*	*D*—H	H⋯*A*	*D*⋯*A*	*D*—H⋯*A*
N2—H2*A*⋯N1^i^	0.88	2.13	2.9948 (17)	166

Symmetry code: (i) 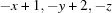.

**Fig. 1.**
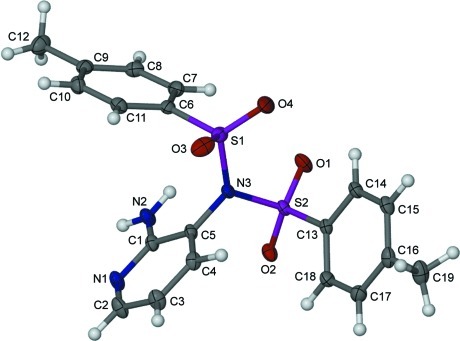
The molecular structure of the title compound showing the atomic numbering scheme. The displacement ellipsoids are shown at the 50% probability level.

**Fig. 2.**
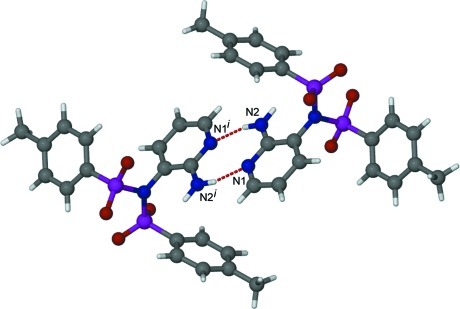
The hydrogen bonding in the title compound, showing the hydrogen bonded ring motif. Intermolecular N—H···N hydrogen bonds are shown as dashed red lines. Symmetry code: -x + 1, -y + 2, -z

## supplementary crystallographic information

### Comment

Aminopyridines and sulfonamides are structural units frequently found in the skeletons of bioactive compounds (Dubey *et al.*, 2000). In this present communication the main aim was to synthesize pyridine-annulated heterocycles by using ring-closing metathesis (RCM) and in which the 2,3-disulfonamide-protected 2,3-diaminopyridine was required as substrate in continuation of previous work in our group combining sulfonamide protecting groups and RCM (Yadav *et al.*, 2011; Morgans *et al.*, 2009). However, in this particular case, when tosyl chloride was utilized in an attempt to 'mono' protect both amino groups on 2,3-diaminopyridine, only the 3,3-ditosyl compound, *N*-(2-amino-3-pyridinyl)-4-methyl-*N*-[(4-methylphenyl)sulfonyl]benzenesulfonamide (I), was isolated. In previous work by our group this behaviour was not observed with 2,3-diaminopyridine or 1,2-diaminobenzene as the substrate (van Otterlo *et al.*, 2004). A literature search indicated that this type of selectivity is not common, see for instance Schetty (1969).

Crystallizing in the space group *P*1, (I) has a single molecule in the asymmetric unit (Fig. 1). In the crystal packing, the molecules associate *via* a centrosymmetric hydrogen bonded dimer with N—H···N hydrogen bonds interacting to form the hydrogen bonded ring motif *R*^2^_2_(8) (Bernstein *et al.*, 1995), Fig. 2. The mean planes passing through the tosyl benzene rings (C6–C11 and C13–C18) form dihedral angles with the aminopyridine ring (N1,C1–C5) of 50.01 (6) and 32.01 (4)°, respectively.

### Experimental

2,3-Diaminopyridine (0.100 g, 0.917 mmol) was dissolved in a mixture of CH_2_Cl_2_ and pyridine (10 ml, 15:1). 4-Methylbenzene-1-sulfonyl chloride (0.524 g, 2.75 mol) was added and the solution was heated, with stirring, to 313 K for 24 h. The solution was allowed to cool to room temperature and washed with dilute HCl (15 ml, 1 *M*) and brine (3 × 15 ml), and then dried over Na_2_SO_4_. After filtration and removal of the solvent under vacuum, the residue was recrystallized from EtOH to give the product as a colourless crystalline material (0.260 g, 68%).

### Refinement

H atoms were positioned geometrically [N—H = 0.88 Å; C—H = 0.95–0.98 Å; with *U*_iso_(H) = 1.2–1.5*U*_eq_(N,C)] and constrained to ride on their parent atoms.

### Crystal data

**Table:**

C_19_H_19_N_3_O_4_S_2_	*Z* = 2
*M**_r_* = 417.49	*F*(000) = 436
Triclinic, *P*1	*D*_x_ = 1.423 Mg m^−^^3^
Hall symbol: -P 1	Mo *K*α radiation, λ = 0.71073 Å
*a* = 8.6343 (15) Å	Cell parameters from 8939 reflections
*b* = 9.6486 (17) Å	θ = 2.3–28.6°
*c* = 12.701 (2) Å	µ = 0.30 mm^−^^1^
α = 111.324 (2)°	*T* = 102 K
β = 90.109 (2)°	Prisms, colourless
γ = 98.097 (2)°	0.25 × 0.25 × 0.25 mm
*V* = 974.2 (3) Å^3^	

### Data collection

**Table:**

Bruker APEXII CCD diffractometer	4644 independent reflections
Radiation source: fine-focus sealed tube, Bruker SMART APEX	4381 reflections with *I* > 2σ(*I*)
Graphite monochromator	*R*_int_ = 0.015
φ and ω scans	θ_max_ = 28.8°, θ_min_ = 1.7°
Absorption correction: multi-scan (*SADABS*; Bruker, 2009)	*h* = −11→11
*T*_min_ = 0.928, *T*_max_ = 0.928	*k* = −12→12
11658 measured reflections	*l* = −17→17

### Refinement

**Table:**

Refinement on *F*^2^	Primary atom site location: structure-invariant direct methods
Least-squares matrix: full	Secondary atom site location: difference Fourier map
*R*[*F*^2^ > 2σ(*F*^2^)] = 0.031	Hydrogen site location: inferred from neighbouring sites
*wR*(*F*^2^) = 0.086	H-atom parameters constrained
*S* = 1.03	*w* = 1/[σ^2^(*F*_o_^2^) + (0.0443*P*)^2^ + 0.5018*P*] where *P* = (*F*_o_^2^ + 2*F*_c_^2^)/3
4644 reflections	(Δ/σ)_max_ = 0.001
256 parameters	Δρ_max_ = 0.47 e Å^−^^3^
0 restraints	Δρ_min_ = −0.35 e Å^−^^3^

### Special details

**Table:**

Geometry. All e.s.d.'s (except the e.s.d. in the dihedral angle between two l.s. planes) are estimated using the full covariance matrix. The cell e.s.d.'s are taken into account individually in the estimation of e.s.d.'s in distances, angles and torsion angles; correlations between e.s.d.'s in cell parameters are only used when they are defined by crystal symmetry. An approximate (isotropic) treatment of cell e.s.d.'s is used for estimating e.s.d.'s involving l.s. planes.
Refinement. Refinement of *F*^2^ against ALL reflections. The weighted *R*-factor *wR* and goodness of fit *S* are based on *F*^2^, conventional *R*-factors *R* are based on *F*, with *F* set to zero for negative *F*^2^. The threshold expression of *F*^2^ > σ(*F*^2^) is used only for calculating *R*-factors(gt) *etc*. and is not relevant to the choice of reflections for refinement. *R*-factors based on *F*^2^ are statistically about twice as large as those based on *F*, and *R*- factors based on ALL data will be even larger.

### Fractional atomic coordinates and isotropic or equivalent isotropic displacement parameters (Å^2^)

**Table:**

	*x*	*y*	*z*	*U*_iso_*/*U*_eq_	
S1	0.69360 (4)	0.75142 (4)	0.30733 (3)	0.02336 (9)	
S2	0.37246 (3)	0.60784 (3)	0.20345 (2)	0.01765 (8)	
N3	0.52302 (12)	0.75248 (12)	0.24209 (9)	0.0186 (2)	
N1	0.47716 (14)	1.06813 (12)	0.15011 (9)	0.0230 (2)	
C5	0.49000 (14)	0.89680 (13)	0.24605 (10)	0.0175 (2)	
C15	0.27102 (16)	0.57171 (14)	0.50145 (11)	0.0232 (3)	
H15	0.3074	0.5249	0.5489	0.028*	
C4	0.43923 (15)	0.99755 (14)	0.34312 (10)	0.0212 (2)	
N2	0.55336 (14)	0.84049 (12)	0.04958 (9)	0.0226 (2)	
H2A	0.5627	0.8686	−0.0090	0.027*	
H2B	0.5743	0.7515	0.0439	0.027*	
O1	0.43589 (12)	0.47071 (10)	0.15842 (8)	0.0268 (2)	
O2	0.26982 (11)	0.64689 (10)	0.13384 (7)	0.02197 (19)	
C13	0.28268 (14)	0.62087 (13)	0.32986 (10)	0.0181 (2)	
O3	0.71981 (12)	0.88223 (13)	0.40887 (8)	0.0331 (2)	
C11	0.92648 (15)	0.91006 (14)	0.23608 (11)	0.0221 (2)	
H11	0.9222	0.9901	0.3065	0.027*	
O4	0.68588 (12)	0.60577 (13)	0.31366 (10)	0.0358 (3)	
C7	0.83292 (15)	0.65472 (14)	0.10759 (11)	0.0231 (3)	
H7	0.7664	0.5614	0.0915	0.028*	
C18	0.16083 (15)	0.70512 (14)	0.36120 (11)	0.0221 (2)	
H18	0.1229	0.7499	0.3129	0.027*	
C6	0.82897 (14)	0.77329 (14)	0.21034 (10)	0.0193 (2)	
C1	0.50712 (14)	0.93365 (13)	0.14787 (10)	0.0189 (2)	
C8	0.93574 (15)	0.67576 (15)	0.02953 (11)	0.0241 (3)	
H8	0.9385	0.5962	−0.0413	0.029*	
C3	0.40867 (17)	1.13551 (14)	0.34406 (11)	0.0257 (3)	
H3	0.3740	1.2072	0.4097	0.031*	
C2	0.43063 (18)	1.16433 (14)	0.24579 (11)	0.0268 (3)	
H2	0.4113	1.2591	0.2463	0.032*	
C16	0.15029 (16)	0.65788 (14)	0.53587 (11)	0.0238 (3)	
C17	0.09552 (16)	0.72267 (15)	0.46422 (12)	0.0249 (3)	
H17	0.0120	0.7799	0.4862	0.030*	
C14	0.33857 (15)	0.55326 (14)	0.39957 (11)	0.0210 (2)	
H14	0.4216	0.4956	0.3774	0.025*	
C19	0.0856 (2)	0.68232 (16)	0.65014 (12)	0.0327 (3)	
H19B	0.0813	0.5900	0.6663	0.049*	
H19C	−0.0202	0.7083	0.6503	0.049*	
H19A	0.1535	0.7646	0.7083	0.049*	
C9	1.03573 (15)	0.81185 (15)	0.05290 (11)	0.0227 (2)	
C10	1.03021 (15)	0.92753 (15)	0.15694 (11)	0.0242 (3)	
H10	1.0986	1.0200	0.1741	0.029*	
C12	1.14802 (17)	0.83002 (19)	−0.03295 (13)	0.0327 (3)	
H12A	1.1896	0.9369	−0.0127	0.049*	
H12C	1.0932	0.7913	−0.1080	0.049*	
H12B	1.2345	0.7738	−0.0343	0.049*	
H4	0.4215	0.9725	0.4113	0.023 (4)*	

### Atomic displacement parameters (Å^2^)

**Table:**

	*U*^11^	*U*^22^	*U*^33^	*U*^12^	*U*^13^	*U*^23^
S1	0.02049 (15)	0.03476 (18)	0.02280 (16)	0.00556 (12)	0.00028 (11)	0.01947 (14)
S2	0.02354 (15)	0.01561 (14)	0.01636 (14)	0.00353 (10)	0.00067 (11)	0.00870 (11)
N3	0.0183 (5)	0.0210 (5)	0.0219 (5)	0.0032 (4)	−0.0003 (4)	0.0144 (4)
N1	0.0351 (6)	0.0178 (5)	0.0182 (5)	0.0031 (4)	0.0017 (4)	0.0094 (4)
C5	0.0201 (5)	0.0174 (5)	0.0174 (5)	0.0008 (4)	−0.0012 (4)	0.0100 (4)
C15	0.0310 (7)	0.0209 (6)	0.0200 (6)	0.0009 (5)	−0.0011 (5)	0.0114 (5)
C4	0.0253 (6)	0.0227 (6)	0.0150 (5)	−0.0010 (5)	−0.0015 (4)	0.0079 (5)
N2	0.0330 (6)	0.0220 (5)	0.0189 (5)	0.0095 (4)	0.0068 (4)	0.0125 (4)
O1	0.0401 (5)	0.0198 (4)	0.0242 (5)	0.0107 (4)	0.0069 (4)	0.0104 (4)
O2	0.0276 (5)	0.0205 (4)	0.0193 (4)	0.0003 (3)	−0.0052 (3)	0.0103 (3)
C13	0.0216 (5)	0.0162 (5)	0.0181 (5)	0.0008 (4)	0.0011 (4)	0.0087 (4)
O3	0.0264 (5)	0.0553 (7)	0.0172 (4)	0.0067 (4)	−0.0019 (4)	0.0129 (4)
C11	0.0253 (6)	0.0201 (6)	0.0211 (6)	0.0042 (5)	−0.0016 (5)	0.0074 (5)
O4	0.0273 (5)	0.0486 (6)	0.0519 (7)	0.0088 (4)	0.0025 (5)	0.0412 (6)
C7	0.0204 (6)	0.0199 (6)	0.0282 (6)	0.0015 (4)	−0.0016 (5)	0.0083 (5)
C18	0.0225 (6)	0.0223 (6)	0.0258 (6)	0.0039 (5)	0.0009 (5)	0.0135 (5)
C6	0.0176 (5)	0.0229 (6)	0.0212 (6)	0.0047 (4)	0.0003 (4)	0.0119 (5)
C1	0.0224 (6)	0.0188 (5)	0.0173 (5)	0.0008 (4)	−0.0001 (4)	0.0095 (4)
C8	0.0218 (6)	0.0245 (6)	0.0236 (6)	0.0061 (5)	−0.0005 (5)	0.0051 (5)
C3	0.0376 (7)	0.0189 (6)	0.0172 (6)	0.0027 (5)	0.0011 (5)	0.0034 (5)
C2	0.0425 (8)	0.0156 (5)	0.0219 (6)	0.0035 (5)	0.0010 (5)	0.0067 (5)
C16	0.0305 (6)	0.0183 (6)	0.0201 (6)	−0.0030 (5)	0.0027 (5)	0.0065 (5)
C17	0.0250 (6)	0.0222 (6)	0.0282 (7)	0.0048 (5)	0.0055 (5)	0.0097 (5)
C14	0.0253 (6)	0.0186 (5)	0.0219 (6)	0.0032 (4)	0.0000 (5)	0.0108 (5)
C19	0.0476 (9)	0.0257 (7)	0.0223 (6)	0.0010 (6)	0.0104 (6)	0.0078 (5)
C9	0.0188 (6)	0.0293 (6)	0.0238 (6)	0.0056 (5)	−0.0001 (5)	0.0135 (5)
C10	0.0244 (6)	0.0217 (6)	0.0272 (6)	−0.0009 (5)	−0.0020 (5)	0.0115 (5)
C12	0.0263 (7)	0.0470 (9)	0.0290 (7)	0.0048 (6)	0.0046 (5)	0.0193 (6)

### Geometric parameters (Å, º)

**Table:**

S1—O4	1.4289 (11)	C11—H11	0.9500
S1—O3	1.4290 (11)	C7—C8	1.3824 (19)
S1—N3	1.6916 (11)	C7—C6	1.3923 (18)
S1—C6	1.7472 (13)	C7—H7	0.9500
S2—O1	1.4257 (10)	C18—C17	1.3884 (18)
S2—O2	1.4292 (9)	C18—H18	0.9500
S2—N3	1.6932 (11)	C8—C9	1.3978 (19)
S2—C13	1.7549 (12)	C8—H8	0.9500
N3—C5	1.4435 (15)	C3—C2	1.3818 (18)
N1—C2	1.3365 (17)	C3—H3	0.9500
N1—C1	1.3487 (16)	C2—H2	0.9500
C5—C4	1.3812 (17)	C16—C17	1.3921 (19)
C5—C1	1.4183 (16)	C16—C19	1.5052 (18)
C15—C14	1.3824 (18)	C17—H17	0.9500
C15—C16	1.3966 (19)	C14—H14	0.9500
C15—H15	0.9500	C19—H19B	0.9800
C4—C3	1.3889 (18)	C19—H19C	0.9800
C4—H4	0.9869	C19—H19A	0.9800
N2—C1	1.3463 (16)	C9—C10	1.3916 (19)
N2—H2A	0.8800	C9—C12	1.5017 (18)
N2—H2B	0.8800	C10—H10	0.9500
C13—C18	1.3905 (17)	C12—H12A	0.9800
C13—C14	1.3962 (16)	C12—H12C	0.9800
C11—C10	1.3875 (19)	C12—H12B	0.9800
C11—C6	1.3891 (17)		
			
O4—S1—O3	119.52 (7)	C11—C6—S1	119.22 (10)
O4—S1—N3	106.78 (6)	C7—C6—S1	119.13 (10)
O3—S1—N3	108.02 (6)	N2—C1—N1	116.60 (11)
O4—S1—C6	110.55 (6)	N2—C1—C5	123.35 (11)
O3—S1—C6	108.89 (6)	N1—C1—C5	120.04 (11)
N3—S1—C6	101.52 (5)	C7—C8—C9	121.25 (12)
O1—S2—O2	120.02 (6)	C7—C8—H8	119.4
O1—S2—N3	108.18 (6)	C9—C8—H8	119.4
O2—S2—N3	103.63 (5)	C2—C3—C4	117.37 (12)
O1—S2—C13	110.28 (6)	C2—C3—H3	121.3
O2—S2—C13	108.76 (6)	C4—C3—H3	121.3
N3—S2—C13	104.73 (5)	N1—C2—C3	124.53 (12)
C5—N3—S1	116.80 (8)	N1—C2—H2	117.7
C5—N3—S2	117.39 (8)	C3—C2—H2	117.7
S1—N3—S2	123.89 (6)	C17—C16—C15	118.80 (12)
C2—N1—C1	118.78 (11)	C17—C16—C19	121.52 (13)
C4—C5—C1	119.84 (11)	C15—C16—C19	119.66 (12)
C4—C5—N3	120.91 (10)	C18—C17—C16	121.10 (12)
C1—C5—N3	119.23 (11)	C18—C17—H17	119.4
C14—C15—C16	121.18 (12)	C16—C17—H17	119.4
C14—C15—H15	119.4	C15—C14—C13	118.87 (12)
C16—C15—H15	119.4	C15—C14—H14	120.6
C5—C4—C3	119.42 (11)	C13—C14—H14	120.6
C5—C4—H4	121.4	C16—C19—H19B	109.5
C3—C4—H4	119.2	C16—C19—H19C	109.5
C1—N2—H2A	120.0	H19B—C19—H19C	109.5
C1—N2—H2B	120.0	C16—C19—H19A	109.5
H2A—N2—H2B	120.0	H19B—C19—H19A	109.5
C18—C13—C14	121.12 (11)	H19C—C19—H19A	109.5
C18—C13—S2	118.79 (9)	C10—C9—C8	118.77 (12)
C14—C13—S2	120.02 (10)	C10—C9—C12	121.34 (12)
C10—C11—C6	118.68 (12)	C8—C9—C12	119.89 (13)
C10—C11—H11	120.7	C11—C10—C9	121.12 (12)
C6—C11—H11	120.7	C11—C10—H10	119.4
C8—C7—C6	118.56 (12)	C9—C10—H10	119.4
C8—C7—H7	120.7	C9—C12—H12A	109.5
C6—C7—H7	120.7	C9—C12—H12C	109.5
C17—C18—C13	118.91 (12)	H12A—C12—H12C	109.5
C17—C18—H18	120.5	C9—C12—H12B	109.5
C13—C18—H18	120.5	H12A—C12—H12B	109.5
C11—C6—C7	121.62 (12)	H12C—C12—H12B	109.5
			
O4—S1—N3—C5	167.92 (9)	O4—S1—C6—C11	−139.70 (10)
O3—S1—N3—C5	38.18 (10)	O3—S1—C6—C11	−6.50 (12)
C6—S1—N3—C5	−76.26 (10)	N3—S1—C6—C11	107.29 (10)
O4—S1—N3—S2	4.12 (10)	O4—S1—C6—C7	42.35 (12)
O3—S1—N3—S2	−125.62 (8)	O3—S1—C6—C7	175.54 (10)
C6—S1—N3—S2	119.94 (8)	N3—S1—C6—C7	−70.67 (11)
O1—S2—N3—C5	155.43 (9)	C2—N1—C1—N2	179.88 (12)
O2—S2—N3—C5	27.00 (10)	C2—N1—C1—C5	−0.38 (19)
C13—S2—N3—C5	−86.95 (9)	C4—C5—C1—N2	−178.83 (12)
O1—S2—N3—S1	−40.86 (9)	N3—C5—C1—N2	−0.11 (18)
O2—S2—N3—S1	−169.29 (7)	C4—C5—C1—N1	1.45 (18)
C13—S2—N3—S1	76.76 (8)	N3—C5—C1—N1	−179.83 (11)
S1—N3—C5—C4	−76.63 (13)	C6—C7—C8—C9	0.90 (19)
S2—N3—C5—C4	88.25 (13)	C5—C4—C3—C2	0.3 (2)
S1—N3—C5—C1	104.66 (11)	C1—N1—C2—C3	−0.8 (2)
S2—N3—C5—C1	−90.46 (12)	C4—C3—C2—N1	0.9 (2)
C1—C5—C4—C3	−1.36 (19)	C14—C15—C16—C17	1.58 (19)
N3—C5—C4—C3	179.93 (11)	C14—C15—C16—C19	−176.71 (12)
O1—S2—C13—C18	−152.71 (10)	C13—C18—C17—C16	0.2 (2)
O2—S2—C13—C18	−19.17 (12)	C15—C16—C17—C18	−1.2 (2)
N3—S2—C13—C18	91.11 (11)	C19—C16—C17—C18	177.04 (12)
O1—S2—C13—C14	30.27 (12)	C16—C15—C14—C13	−0.86 (19)
O2—S2—C13—C14	163.81 (10)	C18—C13—C14—C15	−0.24 (19)
N3—S2—C13—C14	−85.91 (11)	S2—C13—C14—C15	176.71 (9)
C14—C13—C18—C17	0.59 (19)	C7—C8—C9—C10	−0.14 (19)
S2—C13—C18—C17	−176.40 (10)	C7—C8—C9—C12	178.92 (12)
C10—C11—C6—C7	−0.19 (19)	C6—C11—C10—C9	0.98 (19)
C10—C11—C6—S1	−178.09 (10)	C8—C9—C10—C11	−0.82 (19)
C8—C7—C6—C11	−0.73 (19)	C12—C9—C10—C11	−179.86 (12)
C8—C7—C6—S1	177.17 (10)		

### Hydrogen-bond geometry (Å, º)

**Table:**

*D*—H···*A*	*D*—H	H···*A*	*D*···*A*	*D*—H···*A*
N2—H2*A*···N1^i^	0.88	2.13	2.9948 (17)	166

Symmetry code: (i) −*x*+1, −*y*+2, −*z*.
